# Supplementary figures and images for: Disentangled deep generative models reveal coding principles of the human face processing network
Source: PLoS Comput Biol. 2024 Feb 26;20(2):e1011887. doi: 10.1371/journal.pcbi.1011887 (PMC10919870; doi:10.1371/journal.pcbi.1011887)

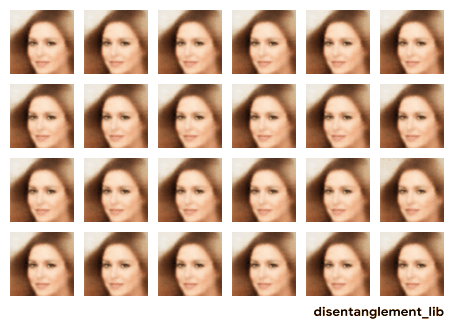

Supplement: S1 Video — Dimensions are varied from -2 to +2, with all other dimensions held constant. Images are model generated. Base image is from the CelebA dataset [20]. (GIF) [file pcbi.1011887.s003.gif]

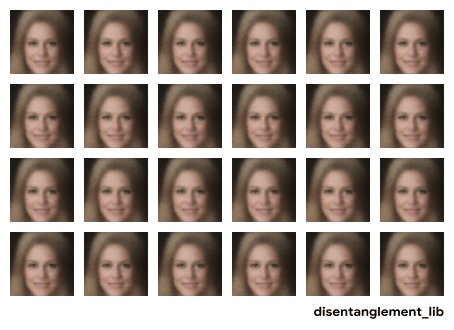

Supplement: S2 Video — Dimensions are varied from -2 to +2, with all other dimensions held constant. Images are model generated. Base image is from the CelebA dataset [20]. (GIF) [file pcbi.1011887.s004.gif]

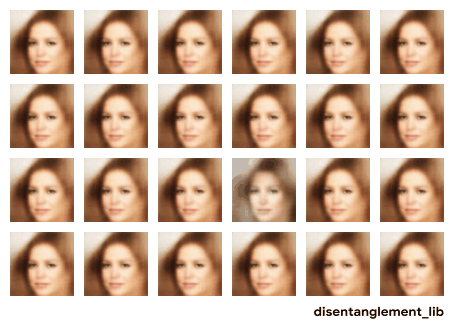

Supplement: S3 Video — Dimensions are varied from -2 to + 2 with all other dimensions held constant. Images are model generated. Base image is from the CelebA dataset [20]. (GIF) [file pcbi.1011887.s005.gif]

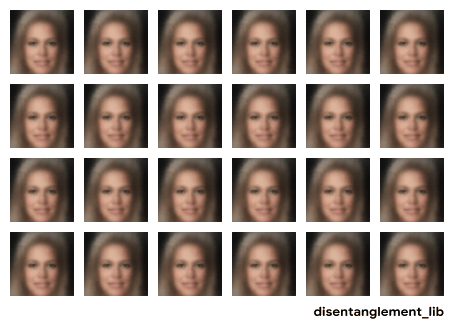

Supplement: S4 Video — Dimensions are varied from -2 to + 2 with all other dimensions held constant. Images are model generated. Base image is from the CelebA dataset [20]. (GIF) [file pcbi.1011887.s006.gif]
