# Supplementary material for: Disentangled deep generative models reveal coding principles of the human face processing network
Source: PLoS Comput Biol. 2024 Feb 26;20(2):e1011887. doi: 10.1371/journal.pcbi.1011887 (PMC10919870; doi:10.1371/journal.pcbi.1011887)
Supplement: Attachment — Submitted filename: DisentangledFaces_response.pdf [file pcbi.1011887.s011.pdf]

Reviewer #1: The authors explored disentangled generative neural networks as a model for human face processing. Identifying a Factor VAE through model selection, the authors employed human raters to interpret the 24 model feature dimensions, finding 16 semantically meaningful. The authors then compared the disentangled model with two conventional models—a VAE without disentanglement and a VGG-based face classification network, showing the disentangled VAE (dVAE) to be a competitive encoding model of human fMRI activity. Furthermore, higher-level dVAE features better matched responses in more anterior face-selective regions. The dVAE also enabled identity decoding from fMRI activity, almost entirely through the eight identity-relevant disentangled features.

Developing high-performance yet interpretable models is an important research goal in visual neuroscience. Disentangled models are a promising direction showing emerging evidence to match neural representations uniquely well (e.g., Higgins et al., Nat. Commun. 2021; Whittington et al., ICLR 2023, arXiv:2210.01768). However, several concerns limit the potential impact of the manuscript.

A key promise of disentangled models is more interpretable features. The manuscript provides limited objective evidence for this supposition. Figure 1 shows examples of feature dimension traversals, which the reader must evaluate subjectively. Reasonable viewers may disagree as to how cleanly disentangled the features are. For instance, it looks to me that the "smile" dimension spuriously correlates with jaw width. Table 1 provides a consensus label for each feature; it would be more informative to report the original annotations given to individual images per rater. The raw data, perhaps quantified with a word embedding metric and evaluated for inter-rater consistency, would provide a more granular and rigorous measure of how interpretable the disentangled features are. The interpretability of the dVAE should ideally be compared to non-disentangled models (i.e., the VAE with beta = 1 and VGG-Face PCs) to establish the advantage of using a dVAE. The authors should also detail how they recruited the human raters. While it is not necessarily a problem for the raters to include the author(s), this should be reported.

Thank you for raising these concerns. We (the authors) were the original annotators and we have now stated this clearly in the results and methods sections. We have added to the supplemental material (TableS2.csv), a csv file with the individual annotation counts across all fifteen images.

The consensus rating for each rater was easy to assign and we used fairly consistent language. Dimension labels sometimes varied across the two labels, but in these cases it was straightforward to match despite language discrepancies (e.g., bangs vs. hairline). We include the labels for the first dimension, which has among the highest inter-rater variability, below for your convenience.

Paper label	Rater 1 labels	Rater 1 counts	Rater 1 consensus		Rater 2 labels	Rater 2 counts	Rater 2 consensus
Lighting / face width	face width	5	Lighting / width		Face width	4	Lighting / face width
	N/A	2			Lighting, Face width	6	
	lighting	6			Lighting	3	
	width	1			Azimuth / lighting	1	
	azimuth	1			Azimuth	1	

We agree that this table is more informative than the majority ratings alone. In making it we also identified an error in our previous methods section about rater agreement (we disagreed on two not one dimensions). The updated Methods section (pg 11, lines 24-34) now reads:

> *The traversal images for each latent dimension were combined into an animated gif. The two authors labeled each dimension in each gif. We first consolidated the annotations for each annotator across images for each dimension by tallying the labels across the 15 face images (see Table S2). We then selected labels where both annotators agreed on the majority of images for our final labels (Table 1). The annotators agreed on 14 out of the 16 labeled dimensions. For the two dimensions that the annotators did not agree on, one annotator assigned a majority label and the other did not. In these two cases (dimension 7 head elevation and dimension 11 hair), we assigned the majority label from one annotator. The remaining 8 dimensions were either not labeled or were not labeled consistently.*

Based on your suggestion, one author also labeled randomized dimensions for the dVAE and VAE models. Of these, 6 were rated as entangled for the dVAE and 19 for the VAE. We believe the other author is so familiar with the different face traversals, they would know which are from the disentangled versus standard VAE, even when presented randomly, so did not have them complete these ratings. We are open to running a separate behavioral experiment to collect annotations from additional participants, if the reviewer feels this is important. In this case, we agree that a word embedding model would be useful to quantify inter-rater agreement across a large proportion of participants.

To now give readers a sense of how the VAE and dVAE dimensions differ, we include visualizations of the dimensions for the same faces as the dVAE (shown in supplemental videos 1 and 2) from the standard VAE as gifs in supplemental videos 3 and 4. Upon visual

inspection, we believe it is clear that unlike those of the dVAE dimensions, most VAE latent dimension traversals depict multiple aspects changing in the example face.

The authors conclude that the disentangled features constitute a decent encoding model of human fMRI data. However, the encoding performance analysis (Fig. 3) likely unfairly represents conventional classification networks. The final fully connected layer of VGG-Face is considerably worse than its intermediate layers (Jiahui et al., biorxiv 2021). VGG-Face is a worse encoding model than ImageNet-trained VGG, which is, in turn, worse than AlexNet and CORnet (Chang et al., Curr. Biol. 2021), both widely used models. It thus remains unclear whether, or by how much, disentangled models suffer an encoding performance cost relative to conventional, less interpretable models for an (as-yet unquantified) gain in interpretability.

Thank you for raising this point. We agree that conventional CNNs could achieve much better encoding performance than the results reported here. While the Jiahui et al. paper shows modest improvements for intermediate model-layers and ImageNet-trained networks, model encoding performance is still quite far from the noise ceiling (though this may be due to the dynamic nature of their stimuli). We now address this point in the discussion (pg. 9 lines 19-27):

> *Interestingly, recent work [10] has shown that object-trained networks do a better job of matching human neural responses to faces than face-trained discriminative networks like VGG-Face tested here. [A1] It is possible that this is due to richer training datasets available for objects than faces [34], which may lead to higher latent dimensionality [35] and improve model match to visual cortex [36]. To date no disentangled models have been successfully trained on such large and diverse datasets. For the fairest comparison, we focused on models trained only on face images. As disentangled models improve at capturing image variation at larger scales, future work can compare disentangled and non-disentangled models on richer, more varied datasets.*

In addition, we expect including more latent dimensions would also improve the VGG model fit. VGG models are trained on more data (millions in Imagenet or VGGFace versus 200k images in CelebA) and have many more tunable parameters than the VAE models we test. This training and inherent dimensionality has been shown to improve encoding performance of visual fMRI data (Elmoznino & Bonner 2022). Unfortunately, our hyperparameter search did not yield a high-dimensional VAE model that was also disentangled (see below). This is an important area for future research that we highlight in our discussion (on pg 9 lines 29-42).

As a result, we believe the most fair test of the role of entanglement is comparing dVAE to the standard VAE. These models provide very similar encoding results, which is the main basis of our claim. We have now clarified this in the discussion (pg 8 lines lines 39-40):

> *This disentanglement improves interpretability without a large degradation in encoding performance, as seen by the similar performance between the disentangled and standard VAEs.*

A third major conclusion of the manuscript is a posterior-anterior gradient of low- to high-level feature representations. This claim should be supported by a quantitative summary of the evidence (Fig. 4). While the overall conclusion likely holds, some presumably low-level features (e.g., “background”) are predictive in FFA, and other high-level features (e.g., “smile”) are also predictive in OFA. There is also considerable inter-subject variation. In quantifying the low-to-high level gradient, the authors would also help the reader by unambiguously defining what low- and high-level features signify. The manuscript seems to suggest high-level features as equivalent to identity-specific, “less changeable” features. However, this interpretation is confusing given that the authors claim no spatial segregation between identity-relevant and -irrelevant features. For another example of the present ambiguities, skin tone may be considered either a low-level visual attribute (because it affects luminance semi-globally) or a high-level one (in being identity-relevant).

Thank you for pointing out these points of confusion. Based on your feedback, we clarified how we operationalized low versus high-level features early in the paper (pg 4, lines 40-45). We define low-level visual features as features that are not specific to faces, which in our case are dimensions 1-7 (lighting, background, image tone, and 3D rotation/elevation). We consider dimensions 8-16 as higher-level dimensions since they are all specific to faces, although as you correctly point out, they are not all identity-specific.

Though as you point out, this distinction is imperfect and we now acknowledge these ambiguities in the results and discussion (pg 8 line 42 - pg 9 line 2):

> *Here we operationalized low versus high-level dimensions as those that are general visual changes (dimensions 1-7) versus face-specific changes (dimensions 8-16), though these are not necessarily identity-relevant. We note though that this distinction is not entirely clear cut as the 3D properties of faces are unique, and it is an open question whether the learned representations for changes in lighting or 3D rotation would generalize to other objects. In addition, properties like skin tone or face-width could also be considered low-level as they affect the overall pixel-level properties of the image.*

We also modified relevant portions of the results section to both clarify and point out the ambiguities in this definition (pg 7):

> *In the OFA, two dimensions, width/lighting and image tone were significantly predictive at the group level. These include lower-level visual changes, which are not face-specific, like lighting and image tone, though we note the first dimension also contains information about face width, which may be predictive of identity (see Discussion). In FFA, one lower-level dimension (background) was significantly predictive at the group level. Additionally, two higher-level, face-specific dimensions, smile and skin tone, and one entangled dimension were also significantly predictive of FFA voxel responses at the group level. When comparing the two ROIs, OFA was significantly better predicted by lighting, image tone, and one entangled dimension, and FFA was significantly better predicted by two entangled dimensions.*

We also ran statistical testing on the predictivity of each dimension in OFA and FFA (see updated Figure 5). We find that OFA is significantly predicted at the group level by two low-level dimensions (lighting and image tone), while FFA is significantly predicted by one low-level dimension (background), two face-specific dimensions (smile and skin tone) and one entangled dimension. We also directly compared predictions across the two ROIs and found that OFA predictivity is significantly greater than FFA in all significant dimensions, while FFA is only greater than OFA in the entangled dimension. We have added these to the results (pg 7, see above, and Figure 5).

Finally, although outside the scope of the current analyses, the authors may consider exploring the "alignment" between disentangled feature dimensions and fMRI responses (Higgins et al., 2021). Aside from interpretability, neuron-to-neuron alignment is perhaps the other main promise of disentangled models. This alignment is the main advantage over conventional models found by Higgins et al. (who did not quantify interpretability). While a negative result would be difficult to interpret given the limited spatial resolution of fMRI, a positive finding would add considerably to the study's impact.

Thank you for suggesting this. We had previously run these analyses and opted to leave them out of the paper, since they yielded negative results, and as you mention, the limited spatial resolution of fMRI makes it difficult to interpret negative alignment results.

We include the results below. The alignment graphs show the average alignment of each single dimension with all voxels in each ROI. As you can see, there is very little difference across models in each ROI. Preliminary analyses indicated this was also true at the voxel level.

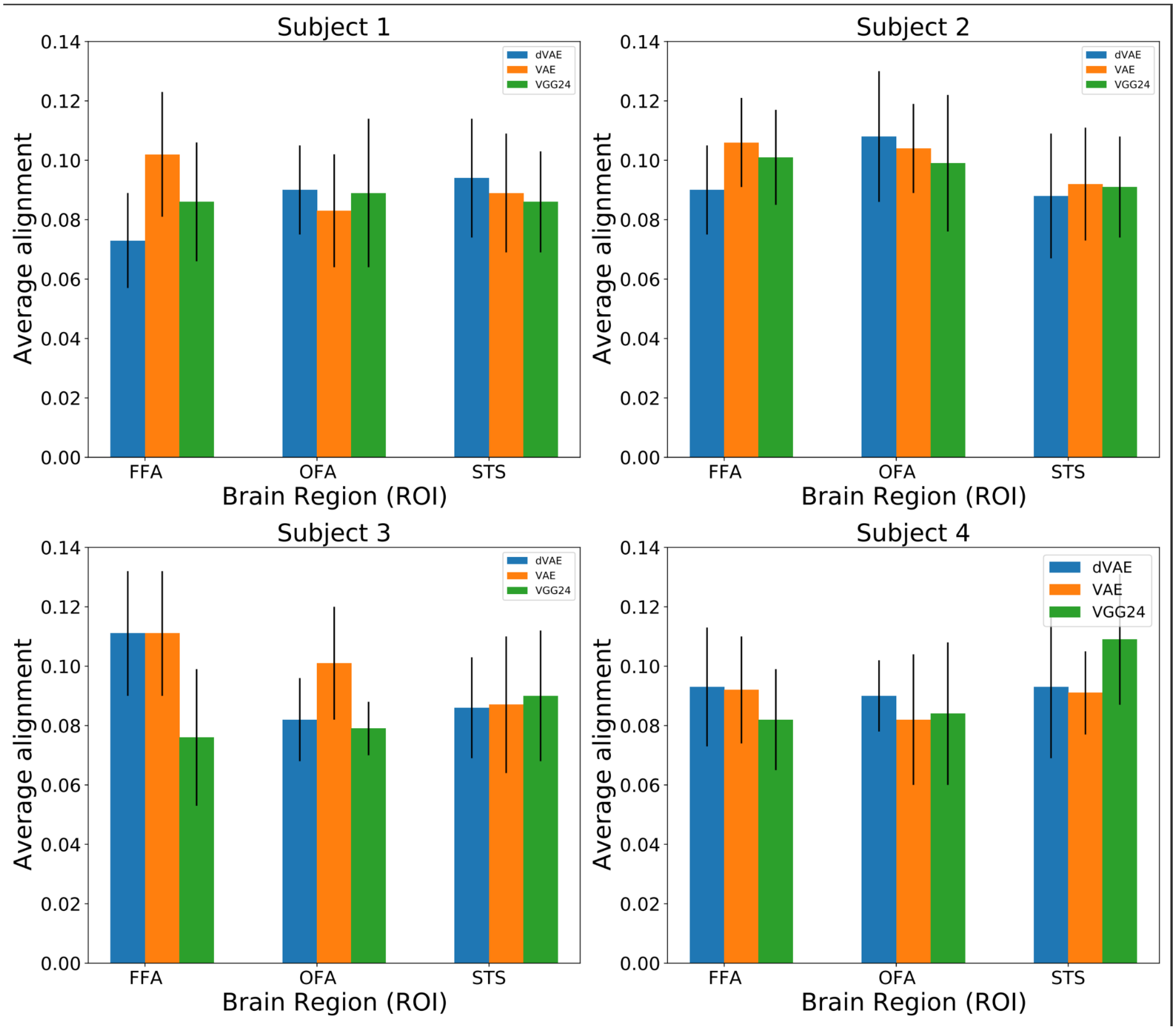

As in Eastwood and Williams 2018 (the paper originally introducing ‘alignment’), we also produced Hinton diagrams for each ROI (shown below). The size of each box represents the relative strength of that dimension, and a single (or few) very large box relative to other small boxes would indicate high disentanglement. Instead, we see several dimensions represented in each ROI, and thus felt the analysis shown in Figure 5 was more robust and easier to follow.

We feel adding these to the paper would not be very informative and distract from the main results, but are open to reconsidering if you think they would enhance the paper.

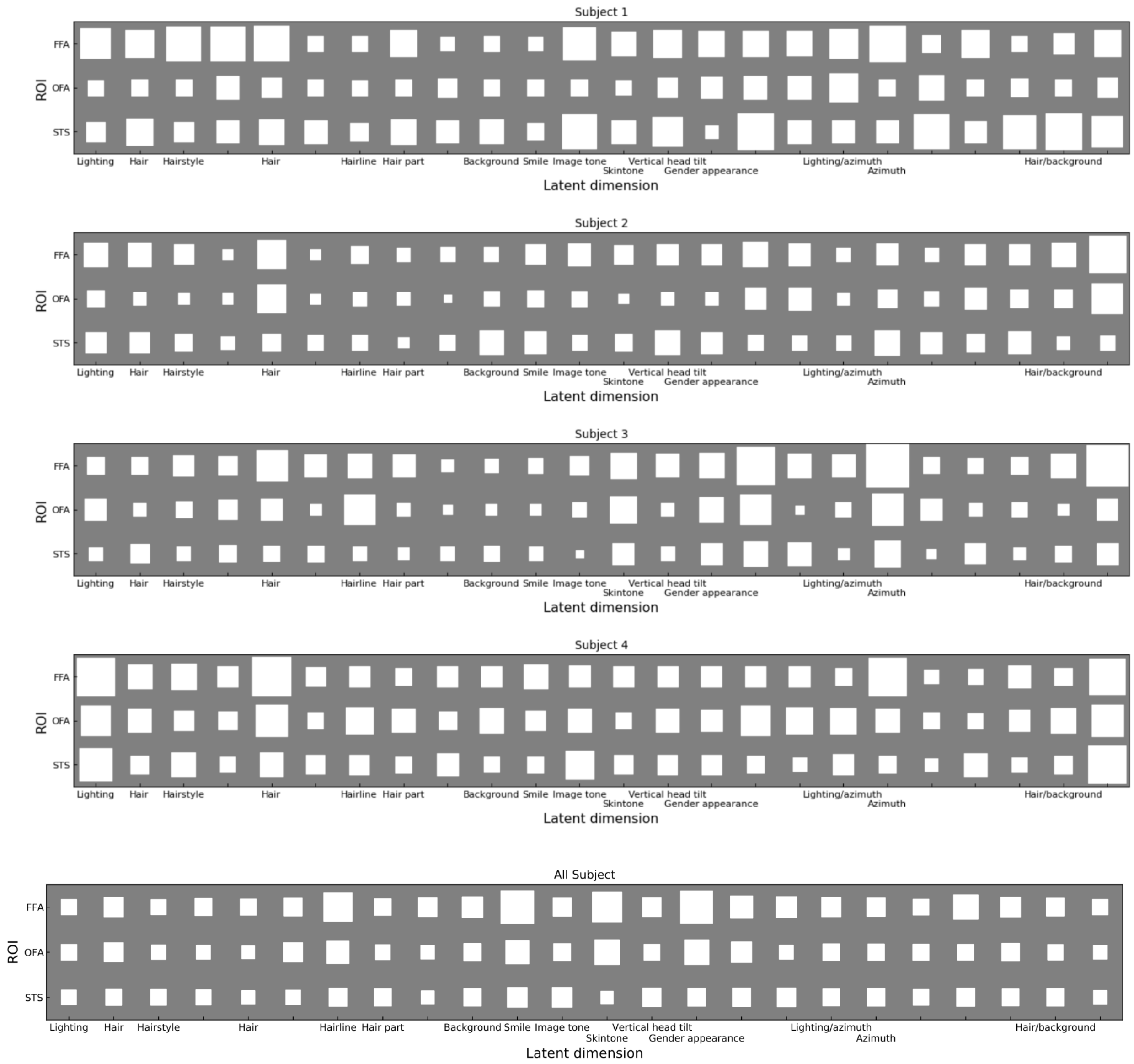

Minor concerns:

I am unsure about the significance of analyzing the information in the “entangled” dimensions. A conventional model has only entangled features, which necessarily contain all the identity information available to the model. The entangled features of a dVAE might simply correspond to the entangled dimensions in conventional models. It seems more remarkable (and thus worth more emphasis) that the disentangled, identity-specific features in a dVAE (i.e., a third of all its dimensions) almost completely explain its ability to decode identity from fMRI.

Thank you for mentioning this point. We agree that the high decoding of the disentangled, identity-relevant features is an exciting result, and we now emphasize it in the discussion (pg 9, lines 6-7):

> *Through decoding analysis, we showed that the disentangled identity-relevant dimensions contain almost all the face identity information in the fMRI signals.*

We still believe the analysis of entangled dimensions is noteworthy, because unlike a standard model with entirely entangled features, these could be the factors of variation that are "left over" after you remove the common factors of variation in the dataset. We try to clarify this in the results and discussion (pg. 8 lines 24-28 and pg. 9 lines 8-10):

> *The role of information contained in the remaining entangled dimensions of a disentangled model is an open question in AI, so we next sought to examine the extent of identity information in these dimensions.*
>
> *In addition to mapping representations of disentangled dimensions across the brain, our approach also allows us to investigate the content contained in the remaining entangled dimensions of the dVAE.*

The authors laudably performed an extensive model search. However, the optimal model corresponded to extreme values in the searched parameter ranges—the lowest gamma and fewest feature dimensions. Should this prompt the authors to consider model instances with even lower gamma and fewer dimensions? Moreover, although beta-VAE underperforms Factor VAE in the UDR selection metric, it would still be useful to analyze beta-VAE to facilitate comparison with prior studies.

Thank you for raising this point that our chosen model corresponded to extreme values on both hyperparameters. The γ values that we searched over were the values used in the paper "Challenging Common Assumptions in the Unsupervised Learning of Disentangled Representations." (Locatello et al., 2018). We have updated the Methods section with this reference.

We originally wrote that we search over γ ϵ [10, 20, 30, 40, 50, 100] when in reality we searched over γ ϵ [0, 10, 20, 30, 40, 50, 100], where γ=0 is a beta-VAE with beta=0. We updated the text to reflect the true range of gamma values (pg 11 lines 6-8):

> *(beta-VAE β ϵ [1, 2, 4, 6, 8, 16], FactorVAE γ ϵ [0, 10, 20, 30, 40, 50, 100] where γ=0 is the same model as a beta-VAE with β=1, a standard VAE). These hyperparameters were selected based on prior work [47].*

While we sought to find the best disentangled model, we were more focused on the potentials of disentangled models as a general class of neural networks for fMRI analysis. We hope that future work by ourselves and others will continue to use better disentangled models than the ones that we used in our paper. We could have looked into models with a smaller latent dimension, but at a certain point the bottleneck becomes so small that we believed encoding performance would suffer dramatically.

In Fig. 3, the asterisks look very similar to the gray scatter dots.

Thank you for pointing this out, we have lightened the gray scatter dots to differentiate them from the black asterisks.

On lines 4 and 11, pg. 6, the author used “significantly.” It is unclear whether this refers to statistical significance and, if so, what test was concerned.

Thank you for pointing this out. We have added group-level statistical testing for the preference mapping and clarified the language on page 7 to clearly indicate which effects are significant versus chance and across the two ROIs.

Line 42, pg. 6: gender appearance should correspond to dark olive color, not green.

We have fixed this reference.

In the Introduction, the authors state that "where [...] face identity is represented is still largely unknown" (line 36). The Discussion suggests that identity and expression coding are still understood to be clear-cut (lines 38-40). The authors should moderate both statements in light of the Duchaine & Yovel review the authors also cite. The review presents preponderant evidence that FFA contains both identity and expression information.

We have edited these statements to read “how face identity…” and “These results add to mounting evidence [Duchaine & Yovel, 2015] challenging a clear-cut…”

The authors used Z-scored p-values to define face-selective ROIs, but p-values do not measure effect size. The raw responses are more appropriate for defining a selectivity index, perhaps with a p-value mask.

Thank you for this suggestion. We and others have used p-values to select voxels for fROIs within individual subjects (e.g., Julian et al., 2012; Isik et al., 2017). We suspect that using a measure of effect size thresholded by p-value would lead to only minor changes in the voxels selected. On the other hand, it would require redoing all of the analyses in the paper, so thus would prefer not to change this definition at this time.

Citation [14] (Higgins et al., “beta-VAE […]”) should be dated 2017, not 2022.

Thank you. We have updated this reference.

Reviewer #2: In the manuscript “Disentangled deep generative models reveal coding principles of the human face processing network”, Soulos and colleagues trained a disentangled deep generative model (dVAE) and used this model as an encoding model for human fMRI data to understand the neural representations of the semantic features of human faces. Using semantically meaningful dimensions from the deep learning models to address the problem of human face representations in the brain is an attractive approach that could integrate AI and neuroscience research. The manuscript is concise and well-written. However, I have reservations about the clarity and robustness of the current results.

Major concerns:
1. The authors trained their model solely on a set of images with limited diversity in face features (CelebA). There's a possibility that the model is biased towards certain dimensions or common image statistics specific to this image set. As numerous face image datasets are readily accessible now, I encourage the authors to broaden their training image set to retrain the model. More importantly, to validate their findings (especially with the neural data), they need to use another deep generative model trained with a different image set.

Thank you for raising this point. Idiosyncrasies of particular image datasets and their impact on learned models are important issues that we tried to highlight in the paper’s discussion. First, we would like to point out that the images the model was trained on (centered and cropped images from CelebA) were not exactly the same as the ones the fMRI participants viewed, which were reconstructed CelebA images from the output of a GAN in the original Van Rullen & Reddy paper. See below for example original face shown to model (left) and GAN-processed face shown to participants (right). These images differ quite a bit in terms of their pixelwise values (e.g., top versus bottom image in the below figure from Van Rullen & Reddy 2019), so hopefully there are not very low-level image statistics driving our results.

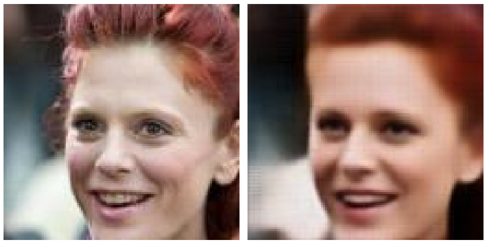

We agree that the dataset still contains higher-level idiosyncrasies that would hamper cross-dataset transfer. We have added the following acknowledgement of this shortcoming to the existing discussion paragraph on dataset limitations (pg 10 lines 30-31):

> *The content of the disentangled dimensions learned by our dVAE, and all other disentangled models, reflects the distribution of features in its training set. CelebA is a dataset of celebrity images which does not reflect the underlying distribution of faces that people see in daily life. In particular, CelebA faces tend to be young adults, white, and smiling at a camera. One example of how this can affect learned representations can be seen in the smile dimension, which is sometimes entangled with wearing sunglasses (Supplementary Video 1), likely reflecting a bias in CelebA that people wearing sunglasses tend to be smiling. More critically, the visual as well as racial and ethnic biases in the dataset likely impact the quality of the learned dimensions [43] and the model's generalization to other datasets. Training models on a more ecologically valid dataset may improve encoding performance by better reflecting the statistics of real-world visual experience.*

We like your suggestion to train the models on a separate natural dataset, but unfortunately don't think it will be successful. Disentangled models are statistical learners whose learned factors of variation come directly from the training data. To date, most disentangled models have been trained on synthetic datasets where the factors of variation are known and well controlled (e.g., full spans of 3D object rotation are shown). To our knowledge, CelebA is the only natural dataset that disentangled models have been successfully trained on, and we think the model would likely break down with larger face datasets (e.g., Labeled Faces in the Wild). For this reason, the two other published papers using VAEs (Van Rullen & Reddy 2019) or dVAEs (Higgens et al., 2021) to model neural responses have each shown participants a subset of the exact same images used to train their models.

In sum, we believe that despite the limitations of the current methods, the pixelwise differences in the fMRI and model images help alleviate concerns about low-level confounds. Further, as disentangled models improve and scale up, we believe the framework presented in our paper will be valuable for testing these models. We've added more discussion of these points on pg 9 lines 23-24 and pg 10 lines 36-37:

> *To date no disentangled models have been successfully trained on such large and diverse datasets.*
>
> *Successfully scaling these models to larger, less constrained datasets is an important, ongoing research challenge. As the quality of models and fMRI data increase, our method can be used to identify new semantically meaningful data dimensions with higher precision.*

2. Many current disentangled dimensions are not face-relevant, including identity-relevant features such as hairstyles. Only a few dimensions (e.g., gender appearance, face width) are purely face dimensions. These disentangled dimensions also fail to separate fine features such as the location, distance, or size of the eyes and nose, which are crucial for face recognition and are represented hierarchically in the brain. Thus, the current dVAE provides a simple and coarse model of portrait images that is heavily influenced by prominent face-irrelevant features.

Thank you for raising these points. We agree that the disentanglement in these models is not perfect, but believe the findings and approach are still valuable. As disentangled models continue to improve, so will this encoding approach (see response to above point).

With regard to the current dimensions, hairstyle, while not strictly speaking a facial feature, is a highly face-relevant dimension both behaviorally to identify faces and it is represented throughout the primate face patch system (Freiwald et al., 2009). Hairstyle is also not a general object-related or visual feature, and thus we believe should be included as a face-relevant dimension (pg 13 lines 17-20):

> *including hair as it offers important cues to facial identity and prior work has shown sensitivity in face-selective voxels [44]*

With regard to fine-grained facial features, there has been much work suggesting these are represented hierarchically in the face patch system (e.g., Freiwald et al., 2009), but more recent work (e.g., Chang and Tsao 2017) suggests a more flexible axis-coding strategy where individual cells are not sensitive to specific identities or facial features. A related recent study with single units also found similarly holistic disentangled features in single cells in the monkey face patch system (Higgins et al., 2021). While our results cannot definitively support the role of one type of feature code over another, we believe this is an interesting open question that we now include in the discussion (pg 10 lines 5-9):

> *On the other hand, the learned dimensions do not seem to represent fine-grained facial features, like the relative position and size of the eyes or nose, which prior work has*

> *found to be represented in the primate face patch system [40]. While it is difficult to say whether this is due to the specific model or dataset we used, it adds to mounting work [41], [42] suggesting a more holistic face-based feature coding.*

3. The authors assert that "Higher-level, identity-relevant dimensions are represented in more anterior face-selective regions." However, this is not clear to me. The dimensions are mixed, and it's challenging to determine which dimensions are “higher-level” ones. Additionally, this study seems to focus more on the three posterior regions (OFA, FFA, and pSTS), so I'm unclear which regions are considered “anterior” and how these anterior regions were defined. This part of the results needs a clear definition and rigorous analysis to compare the representations between “higher vs lower” level dimensions and “anterior vs posterior” face-selective regions to substantiate the conclusion.

Thank you for pointing out these points of confusion. First, we define low-level visual features as features that are not specific to faces, which in our case are dimensions 1-7 (lighting, background, image tone, and 3D rotation/elevation). We consider dimensions 8-16 as higher-level dimensions since they are all specific to faces, although as you correctly point out, they are not all identity-specific. We have aimed to clarify this in the results section (pg 4 lines 40-45 and pg 7 lines 5-8). Though like you point out, this distinction is imperfect and we now acknowledge these ambiguities in the discussion (pg 8 line 42 - pg 9 line 2):

> *Here we operationalized low versus high-level dimensions as those that are general visual changes (dimensions 1-7) versus face-specific changes (dimensions 8-16), though these are not necessarily identity-relevant. We note though that this distinction is not entirely clear cut as the 3D properties of faces are unique, and it is an open question whether the learned representations for changes in lighting or 3D rotation would generalize to other objects. In addition, properties like skin tone or face-width could also be considered low-level as they affect the overall pixel-level properties of the image.*

For the ROIs considered, FFA is more anterior to and considered to be a later stage of face processing than OFA, which is where our main statistical comparisons focus (see results pg 6 and updated Figure 5). While we do not have a reliable parcel to define more anterior ventral regions, several individual subjects have reliable responses in anterior ventral voxels, which we discuss in the whole brain results (pg 6 lines 41-45). Due to the low reliability along the STS, we focus primarily on these ventral regions.

4. This study primarily relies on neural data from four participants, and the inconsistent prediction performance among these participants raises concerns. There are significant inter-subject variations in the results, and with only four data points, it's challenging to

gauge the robustness of the results and their generalizability across individuals. I have significant reservations about the small number of participants and strongly recommend the authors to include another dataset or more participants to demonstrate the reliability and generalizability of their findings.

We used a previously published dataset (Van Rullen & Reddy 2019) for all analyses in our paper, and thus it is unfortunately infeasible for us to collect data from additional participants. While we acknowledge that dataset noise is a limitation of the current results (pg 10 lines 37 - 39), recent empirical and theoretical work in cognitive neuroscience has highlighted the benefits of using a small-n, condition-rich design over more traditional approaches that that show a small number of stimuli to many individual subjects (Naselerais et al., 2021, Fedorenko et al., 2010). We now explain this justification when we introduce the fMRI dataset (pg 5 lines 18-20).

5. I am concerned about the capability of human fMRI data to work with deep neural network models, given that fMRI data lacks the spatial resolution necessary to accurately map the features. The correlations shown in this study are also not particularly high, especially considering the strong effect from non-identity low-level features such as the background. The authors have briefly touched upon this in the discussion, but expanding on this point could help readers understand the limitations of this study.

We have expanded this portion of the discussion (pg 10 lines 18-21):

> *Even at the voxel level, it may not be possible to see evidence for single disentangled dimensions. It remains an open question whether, and at what spatial scale, the primate face network is disentangled or shows exact correspondence to the dimensions learned by dVAEs across the visual system.*

However, one major advantage of fMRI for our purposes, is the ability to look at information across all face-selective voxels across the brain (versus the limited sampling of intracranial recordings). Several recent fMRI-DNN papers have shown high correspondence between voxels and models with enough data (e.g., the Natural Scenes Datasets and many recent modeling papers that have come out of that). Ideally in the future new advances in neuropixel technology and higher resolution fMRI will provide data with both high spatial resolution and broad scale coverage to fully test brain-model correspondence.

Minor concerns:
1. The method of calculating accuracies in Figure 6 is unclear. From the methods section, it appears that the decoding was based on the correlations between the predicted and actual

latent dimensions . However, it is not clear how the authors converted these correlation values into accuracy values. This process requires further explanation.

We have revised the text in section "M7. Identity Decoding" to make the procedure more clear. It now reads:

> *If the correlation between the predicted latent dimensions and the true latent dimensions was larger than the correlation between the predicted latent dimensions and the foil, that indicated that the identity was correctly decoded. This pairwise comparison was repeated with every test image as the ground truth, and every other test image as the foil for a total of 20*19=380 samples per subject. The total accuracy was the number of correct identity decoding divided by 380. Please let us know if the additional details sufficiently address the procedure.*

2. There are no error bars in any of the plots, possibly due to the large variability across individuals when the study only includes four participants. If more participants can be included, it is recommended that error bars are added to aid understanding of the bar plots.

Thanks for this suggestion. We chose to include dots for the four subjects instead of error bars, to be maximally informative, given the small sample size. (Please see above response to point 4 for the explanation of our small sample size.) We are open to including error bars showing standard error instead, but we believe this will obscure the inter-subject variability.
