# Supplementary material for: Disentangled deep generative models reveal coding principles of the human face processing network
Source: PLoS Comput Biol. 2024 Feb 26;20(2):e1011887. doi: 10.1371/journal.pcbi.1011887 (PMC10919870; doi:10.1371/journal.pcbi.1011887)
Supplement: Attachment — Submitted filename: Review_Response.pdf [file pcbi.1011887.s012.pdf]

Reviewer 1

I appreciate the revisions the authors made. The results are more straightforward to evaluate and better contextualized.

My main comments this time are to tighten the conclusions. The authors claim the following in the abstract:
1. The majority of learned latent dimensions in [the dVAE] are interpretable by human raters
2. These latent dimensions serve as a good encoding model for human fMRI data
3. [There is] a gradient from low- to high-level face feature representations along posterior to anterior face-selective regions
4. A decoding analysis confirms that the model separates identity-relevant and -irrelevant information
5. [There is] no spatial segregation between identity-relevant and -irrelevant face features
6. The few "entangled" (uninterpretable) dimensions
6a. match responses across the ventral stream
6b. carry significant information about facial identity

I think claims 1, 2, and 4 are now reasonably well supported. I appreciate the more direct description of interpretability ratings and the addition of Table S2. I agree that adding participants for rating is not essential to the paper's more interesting results on brain decoding.

Claim 3 is still unclear. Part of the issue is the wording—I think 'gradient' does not aptly describe comparisons between two ROIs. Moreover, while per-ROI statistics identify two face-specific (i.e., 'high-level') dimensions in FFA, between-ROI statistics only show statistically significant differences in an entangled feature in FFA, which does not directly support the claim of a gradient from low- to high-level features. (I do appreciate the addition of between-ROI statistics.) Another issue is that the evaluation of the voxel-wise results is purely qualitative, and I am unsure to what degree they support the claim of a gradient. Fig. S4 was hard for me to read. A different color map would help, giving high-level and low-level two families of colors (e.g., warm and cold). (The current color map puts features in groups of 2 that are irrelevant to the paper's claims, distracting, and require repeated references to the figure key.)

Thank you for your positive feedback and for these suggestions, we agree and address the term 'gradient' below. We have now updated the color bar for Table 1, Figure 5, and Figure S3 and S4 so that low-level features are represented with darker colors and pastels are used for high-level/face-specific features. In making this update, we noticed a few dimensions

were mislabeled in Figure S4 (though not in Figure 5 or elsewhere in the text). This error also did not affect the results text.

It is unclear what evidence directly supports claims 5 and 6a. Particularly for claim 5, the word segregation never appears in the main text. Are claims 5 and 6a based on the same evidence as claim 3 (i.e., Figs. 5 and S4)? Are both claims rigorously testable? I.e., what results, ideally quantitative ones, would support or reject the respective claims?

In claim 6b, it again helps to specify whether 'significant' refers to statistical significance. I found no statistical tests associated with Fig. 6. Is claim 6 really about 'above-chance' decoding? It helps to indicate chance in Fig. 6 (50% if I understand correctly).

We removed the use of the term ‘significant’ in the introduction related to the claims in Fig 6. We also updated Fig 6 and its caption to better indicate chance performance.

I think suitably re-wording claims 3, 5, and 6 will not detract from the paper's significance and requires no additional analysis, although additional analysis may further strengthen claim 3.

Thank you for pointing out these remaining discrepancies. Based on your suggestions we have tightened the wording around claims 3,5, and 6 in the abstract and Discussion. In particular, we have removed the word “gradient” which we agree was confusing, and also removed the phrases “segregated” and “throughout the ventral stream”. The abstract now reads:

> We find that low- and high-level face feature features are represented in posterior and anterior face-selective regions, respectively, corroborating prior models of human face recognition. Interestingly, though, we find identity-relevant and irrelevant face features across the face processing network. Finally, we provide new insight into the few "entangled" (uninterpretable) dimensions in our model by showing that they match responses in the ventral stream and carry information about facial identity.

We have made similar changes to the first paragraph of the discussion.

Minor comments:
The Discussion explains well why the authors chose 24 latent dimensions (lines 10.31-10.40). A preview of this is due when this parameter was first introduced (line 4.31). The number of model latent dimensions is relevant. A different choice can potentially affect the conclusions about interpretability and the two classes of features (identity-relevant or not).

We have updated this to read:

> Based on a hyperparameter search over previously published model architectures, number of latent dimensions, and model-specific disentanglement parameters to maximize disentanglement (see Methods M1),

Why do the authors distinguish high- and low-level features vs. identity-relevant and -irrelevant features? The reason is implicit in some places (e.g., Fig. 6) but not in others, and juxtaposing the two categorization systems was confusing (e.g., in the abstract and on page 7). It would help the reader to explain why each analysis used either categorization and emphasize the subtle difference between the two since only one feature distinguishes them (dim 13. 'smile').

Thank you for pointing out this confusion. We have added a note to clarify this distinction when we introduce the concept of identity-relevant features (pg.8, lines 15-19).

> *Note that in our set, identity-relevant dimensions include all face-specific features identified above, with the exception of smile, which is not relevant to identity.*

Line 4.39, 'agreed on': This phrasing is confusing. The Method (and reviewer response) is unambiguous—the authors agreed on 14 dimensions, and the other two dimensions were interpretable to one rater and conceded by the other.

We have clarified this point in the Results. It now reads:

> Out of the 24 latent dimensions, the authors agreed on semantic labels for 16 (14 unanimously and two for a single rater, See Methods M2, Table 1)

Line 5.13, 'correlated': Do the latent dimensions correlate or have similar geometry? The word 'correlated' is confusing because it could mean individual features are correlated, which would contradict the result that dVAE features are more disentangled and interpretable.

Thank you for pointing this out. We meant the correlation of the latent space or as you say "similar geometry". We have updated this section:

> While the dVAE and VAE latent dimensions shared a similar geometry (CCA r = 0.92), the dVAE and VGG latent spaces were only moderately correlated (CCA r = 0.52), suggesting that discriminative versus generative training frameworks result in different face representations.

In Fig. 5, it helps to annotate the dimensions showing significant differences between OFA and FFA.

Done

Lines 7.37–7.40: The posterior voxels are not that clear. Where posteriorly can I see background (oranges) and image tone (pale blue)?

These are both around the OFA in more lateral posterior regions as well as in the more posterior portions of the ventral surface (including posterior portions of FFA). We believe background and lighting are the most salient dimensions so have updated the text accordingly. They are now colored dark blue and orange.

Lines 8.24-26, 'The role of information contained in the remaining entangled dimensions of a disentangled model is an open question in AI': I'm still unconvinced this is a significant question in AI. I must be less familiar with the literature than the authors. Thus, the authors can help by discussing or citing work that discusses why residual entangled dimensions are an interesting and important open question in AI.

Thank you for pointing out that we need to strengthen the case for interpreting entangled dimensions in disentangled models. While the content of entangled dimensions is a somewhat specialized topic, it relates more generally to model interpretability, especially for models that use face images to make significant decisions such as criminal sentencing. Most of the prior work on disentangled models has focused on toy datasets where the data is generated according to an underlying generative process that the model learns to invert, so we believe our application is particularly relevant to computer vision given the use of our unconstrained dataset.

We've clarified this point and added a key reference to the discussion: Zhou et al (2021). In this paper, the authors point out while discussing supervised vs unsupervised disentanglement metrics: "However, datasets in the wild, which CelebA veers closer to, differ in this respect. We do not know the complete set of factors of variation for CelebA's human faces, and the attributes provided in the metadata are a subset at best." We hope our work advances the interpretability of these models (and hidden factors of variation in their training datasets) by shedding light on the content of entangled dimensions. We have sought to clarify this in the discussion (page 9 lines 15-18):

> *The nature of learned representations in models trained on naturalistic data is an open question in AI (Zhou et al 2021). Our approach also allows us to investigate the content contained in the remaining entangled dimensions of the dVAE.*

Line 9.29, 'he combination': typo.
We have fixed this typo

Lines 10.16–19: Given that the authors have conducted a direct (preliminary) analysis on alignment, it is well to mention it here. I agree with the authors about not including the relevant figures in the reviewer response as supplementary figures, but only because the plots show no interpretable differences, not because the analysis itself is distracting.

We now briefly mention this preliminary analysis in this section:

> They also demonstrate a high degree of disentanglement in the macaque neurons by showing a strong correlation between model disentanglement and alignment with IT neurons. Interestingly, in preliminary analyses (results not shown), we did not find high alignment between single latent dimensions and our fMRI data. It is worth noting that only a handful of neurons in the macaque data show high alignment with single disentangled dimensions. Perhaps unsurprisingly given the lower spatial resolution of fMRI, we do not see the same high disentanglement in our data as evidenced by the fact that each region is well predicted by multiple latent dimensions. Even at the voxel level, it may not be possible to see evidence for single disentangled dimensions.

Finally, thank you for your attention to our public code. The lack of a README was an oversight on our part. Instructions were added to the publicly available code on github. We uploaded the beta weights and model weights to an OSF repository for replication.

Reviewer 2

The authors have successfully addressed most of my questions and concerns. The only two points I would like to discuss with the authors are related to previous major concerns 3 and 5.

1. My understanding remains unclear regarding how the authors define the anterior and posterior regions. I am skeptical about relying solely on a few ventral regions to draw conclusions about the gradient. I would recommend that the authors discuss this limitation in the discussion section.

Thank you for your helpful feedback and for raising this concern. We agree that there is not strong support for such a “gradient” and have removed this term from our abstract and conclusion. Based on your suggestions, we also mention the fact that our conclusions are largely limited to the ventral stream due to low-reliability in the STS and more anterior regions in the first paragraph of the discussion.

> We note however, that these conclusions are largely based on analyses in OFA and FFA due to low reliability in lateral and anterior regions. An interesting question for future work is how disentangled model models match representations in the extended face processing network.

2. Research involving human fMRI data and deep neural network models appears to produce more inconsistent results when modeling faces compared to other object categories, such as scenes from natural datasets. I would be interested in the authors' perspective on this discrepancy.

This is a very interesting question that we believe is related to the point we raise in the Discussion about the existence of richer and more varied training datasets for object and scene recognition than face recognition. We have expanded that section to speculate on why we think there is worse match between DCNNs and face versus object/scene selective regions on pg 9, lines 26-30:

> Interestingly, recent work [10] has shown that object-trained networks do a better job of matching human neural responses to faces than face-trained discriminative networks like VGG-Face tested here, though in general face-selective responses are not as well explained by DCNNs as object- and scene-selective regions. It is possible that this is due to richer training datasets available for objects than faces [34], which may lead to higher latent dimensionality [35] and improve model match to visual cortex [36]. To date no disentangled models have been successfully trained on such large and diverse datasets.
